# Supporting Pain Self-Management in Patients With Cancer: App Development Based on a Theoretical and Evidence-Driven Approach

**DOI:** 10.2196/49471

**Published:** 2023-10-09

**Authors:** Asma A Abahussin, Robert M West, David C Wong, Lucy E Ziegler, Matthew J Allsop

**Affiliations:** 1 Department of Biomedical Technology College of Applied Medical Sciences, King Saud University Riyadh Saudi Arabia; 2 Leeds Institute of Health Sciences School of Medicine, University of Leeds Leeds United Kingdom

**Keywords:** pain, cancer, behavior change, capability, opportunity, motivation, and behavior model, COM-B model, Behavior Change Wheel, BCW, mobile health, mHealth, app, pain self-management, evidence-based, intervention design, theory

## Abstract

**Background:**

To inform the development of an intervention, it is essential to have a well-developed theoretical understanding of how an intervention causes change, as stated in the UK Medical Research Council guidelines for developing complex interventions. Theoretical foundations are often ignored in the development of mobile health apps intended to support pain self-management for patients with cancer.

**Objective:**

This study aims to systematically set a theory- and evidence-driven design for a pain self-management app and specify the app’s active features.

**Methods:**

The Behavior Change Wheel (BCW) framework, a step-by-step theoretical approach to the development of interventions, was adopted to achieve the aim of this study. This started by understanding and identifying sources of behavior that could be targeted to support better pain management. Ultimately, the application of the BCW framework guided the identification of the active contents of the app, which were characterized using the Behavior Change Technique Taxonomy version 1.

**Results:**

The theoretical analysis revealed that patients may have deficits in their capability, opportunity, and motivation that prevent them from performing pain self-management. The app needs to use education, persuasion, training, and enablement intervention functions because, based on the analysis, they were found the most likely to address the specified factors. Eighteen behavior change techniques were selected to describe precisely how the intervention functions can be presented to induce the desired change regarding the intervention context. In other words, they were selected to form the active contents of the app, potentially reducing barriers and serving to support patients in the self-management of pain while using the app.

**Conclusions:**

This study fully reports the design and development of a pain self-management app underpinned by theory and evidence and intended for patients with cancer. It provides a model example of the BCW framework application for health app development. The work presented in this study is the first systematic theory- and evidence-driven design for a pain app for patients with cancer. This systematic approach can support clarity in evaluating the intervention’s underlying mechanisms and support future replication.

## Introduction

### Background

In patients with cancer, pain is one of the most devastating symptoms throughout the cancer stages, during which it increases in prevalence throughout and beyond cancer treatment [1,2]. Approximately one-third (31.8%) of the patients with cancer who experience pain do not receive pain medication proportional to their pain intensity [3]. Uncontrolled pain has a significant disabling effect on the daily activities and emotions of a patient with cancer, reducing their quality of life [4,5]. Evidence suggests that empowering patients with cancer and endorsing pain self-management have significant benefits in optimizing pain management [6-8].

A systematic review revealed that mobile apps have been increasingly reported as delivering health behavior change interventions and showing promising results [9]. Significantly, evidence shows that self-management interventions for patients delivered via mobile apps are effective compared with self-management interventions delivered via traditional methods or along with usual care in chronic conditions such as diabetes and cardiovascular diseases [10]. However, there is a paucity of evidence exploring the use of mobile health (mHealth) apps for improving the care of patients with cancer and particularly for supporting pain management, as also observed by Boceta et al [11].

According to the UK Medical Research Council (MRC) guidelines for developing complex interventions, a solid theoretical understanding of how an intervention causes change is required to inform its development [12]. Indeed, the use, particularly extensive use, of theory and multiple behavior change techniques (BCTs) in internet- and mHealth-based interventions was associated with significant levels of effectiveness [13,14]. However, contrary to this guidance, evidence has shown that the use of theory was either not mentioned or not explicitly discussed regarding how it was applied to drive the design and development of mobile apps, particularly apps for people with cancer [14,15]. Many reviews on pain-related apps in general have confirmed that the reviewed apps lacked both theoretical rationale and evidence-based features and strategies [16-18]. The reviews concluded with highlighting the need to consider the theoretical and evidence-based foundation for designing and developing pain apps to better support patients’ pain self-management.

In relation to interventions for pain self-management for patients with cancer in particular, systematic reviews and meta-analyses have shown that such interventions are effective in supporting better pain management [7,8,19-21]. However, the studies did not reveal which intervention component or combination of components was the most effective. Koller et al [8] and later Howell et al [7] reviewed the structure and content of interventions designed to improve patients’ self-management of cancer pain, aiming to identify the efficacy of different components. Despite the detailed description of the interventions’ components provided by the 2 reviews, the most efficacious component or group of components could not be determined. As discussed by the authors, this was related to the heterogeneity in the designs of the reviewed studies and the variability in the number of structure and content components of the interventions. Therefore, there is a need for interventions to be designed and developed considering a systematic approach that makes it possible to characterize interventions. Certainly, characterizing interventions by standardized and well-defined BCTs, which are active components, is required to achieve two important aspects: (1) enable tracking mechanisms subsidizing effectiveness across interventions and (2) enable the replication and development of effective interventions [22-25]. In addition, it seems that there is a variation in perceiving the term *pain self-management*. This has led to heterogeneity in the focus and content of pain self-management support interventions for patients with cancer. Indeed, Howell et al [7] emphasized the need for consensus when defining the essential components of cancer self-management to ensure the consistent and effective delivery of such interventions.

### Insight Into Pain Self-Management Concept

A review of Cochrane reviews of the self-management of chronic condition interventions stated that, in practice, the term “self-management” has been used to describe both simple and complex interventions aimed to empower individuals to manage their own health. Such interventions focused on educating patients about their condition and providing them with basic skills to manage their disease symptoms daily [26]. The latter is required to build self-efficacy, which is deemed a key element attributed to behavior change and health outcomes [27]. It refers to the belief in one’s own abilities to establish and execute the courses of action required to achieve specified goals [28].

Pain self-management interventions for patients with cancer have been described as complex interventions because they need to incorporate several interacting components, reflecting the complexity of cancer pain [7,12,29]. A recent review has addressed the need for defining these components and detailed the concept of self-management of cancer pain [30]. Consequently, the latter has been defined as “the process in which patients with cancer pain make the decision to manage their pain, enhance their self-efficacy by solving problems caused by the pain, and incorporate pain-relieving strategies into daily life, through interactions with health-care professionals” [30]. Thus, five attributes were identified for cancer pain self-management as follows: (1) interactions with health care professionals (HCPs), (2) decision-making for pain management, (3) pain-related problem-solving, (4) self-efficacy, and (5) incorporating strategies for pain relief into daily life. These attributes were suggested to be used as modules of nursing practice promoting patient self-management of cancer pain [30].

### Behavior Change Theories and Models

There are many theories and models for behavior change, such as the theory of reasoned action [31] and the theory of planned behavior [32]; however, there has been a lack of guidance and rationale for selecting a specific model or theory for a particular context [33,34]. In addition, many of the models or theories share or have overlapping constructs, making it difficult to know how to select and apply theories [35]. Behavior change intervention development frameworks, such as intervention mapping [36] and the BCT taxonomy developed by Abraham et al [37], contribute to translating theory into practice [34]. Nineteen existing frameworks, including the aforementioned ones, were identified in a systematic review study, evaluated in terms of usefulness, and criticized in regard to ≥1 of 3 aspects: not being linked to an overarching behavior model, being conceptually incoherent, or being uncomprehensive in terms of offering designers the full range of options to change behavior [34]. The Behavior Change Wheel (BCW) framework was constructed to overcome these limitations by synthesizing the common features of the frameworks. It provides a step-by-step method for systematic and theory-based design and the development of behavior change interventions that can be characterized by BCTs [34,38].

The BCW is based on the capability, opportunity, motivation, and behavior (COM-B) model that suggests that interaction among 3 components, namely capability (C), opportunity (O), and motivation (M), produces behavior (B) that, in turn, influences them [34,38]. Thus, changing behavior requires changing ≥1 of these components. Each component is subdivided into 2 types as follows: physical and psychological capability, social and physical opportunity, and reflective and automatic motivation. The BCW is designed to drive intervention designers into building behavioral analysis to understand the targeted behavior using this model. The analysis helps identify what is missing and what needs to change for a desired behavior to occur and contribute to solving a problem. Next, the BCW allows designers to identify which of 9 possible intervention functions could best bring about change. Moreover, it supports the selection of the best policy category, if required, for delivering the intervention from 7 specified categories. It then suggests specifying the content of the intervention through selecting the appropriate BCTs that best serve the identified intervention functions. The BCTs, or the “active ingredients of an intervention” as described by the BCW, can be selected from the Behavior Change Technique Taxonomy version 1 (BCTTv1), which is the international consensus taxonomy of 93 evidence-based BCTs clustered within 16 categories [25]. The BCW framework also provides guidance in specifying the appropriate mode of delivery to implement an intervention, if needed [38].

The BCW is an increasingly applied framework for designing and developing behavior change interventions in various health-related problems and contexts [39-42] (some interventions were delivered through apps [43-46]). However, to the best of our knowledge, the BCW has never been used in the context of supporting pain self-management for patients with cancer. Indeed, Koller et al [29] used only the BCTTv1 to code and describe their “ANtiPain” intervention for patients with cancer in their pilot randomized controlled trial study. Therefore, this study aims to design a pain self-management app for patients with cancer and specify its active features by following a detailed application of the BCW. This is the first systematic theory- and evidence-driven design for an app in this context.

## Methods

### Ethical Considerations

No ethical approval was required for this study, according to the Research Ethics Committee at King Saud University. The study was based on reviewing the literature and applying a theoretical framework, and no human or animal subjects were involved.

### Overview

**Figure 1:**
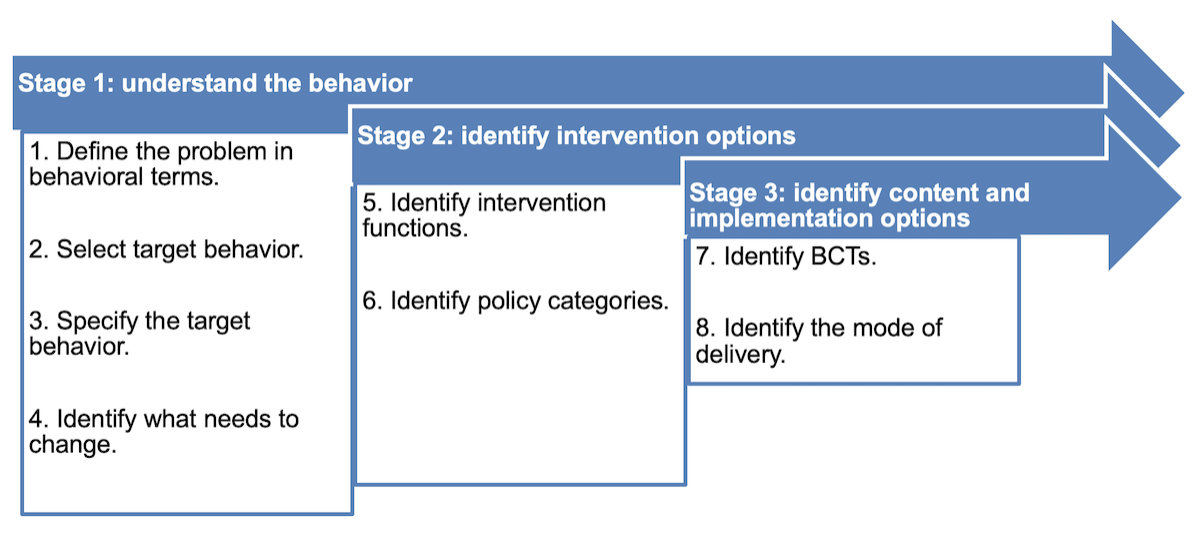
Behavior change intervention design process. BCT: behavior change technique.

According to the BCW framework, there are 8 steps grouped into 3 stages for designing behavior change interventions (Figure 1). The steps can be conducted with flexibility according to the need and context for each individual study [38]. For this study, they were adapted and conducted as detailed in the following subsections.

### Stage 1: Understanding the Behavior and What Needs to Change

#### Step 1: Defining the Problem in Behavioral Terms

This step aims to define the problem of inadequate pain management for patients with cancer in behavioral terms. This involved considering all behaviors from individuals, groups, or populations that potentially contribute to the problem. To accomplish this step, we relied on reviewing the literature to understand the barriers and facilitators of adequate cancer pain management. The MEDLINE electronic database was searched using a combination of terms, including *barriers*, *facilitators*, *pain management*, and *cancer*, and synonyms of these terms.

#### Step 2: Selecting Target Behavior

The aim of this step was to select 1 target behavior to be addressed by the intervention because it is recommended limiting the intervention to just 1 or a few behaviors to increase the intensity and effectiveness of the intervention [38]. The target behavior was selected based on evidence discussed in the literature regarding factors that hinder effective pain management [6-8]. As the app is oriented to the patient’s use in the home setting, we focused on pain management behaviors attributed to patients. The behavior that showed the strongest supporting evidence for better pain management was selected as the target behavior.

#### Step 3: Specifying the Target Behavior or Behaviors

In this step, the target behavior was specified in terms of the context in which it occurs, including who performs it, what needs to be performed to achieve the desired change, and when and where it is performed.

#### Step 4: Identifying What Needs to Change

This step aims to identify the determinants for the target behavior specified from the previous steps, which involved pain self-management. A behavioral diagnosis was conducted to identify what needs to be changed in relation to the COM-B components for the selected target behavior to be performed. This means exploring the barriers to, and facilitators of, patients’ capability, opportunity, and motivation to perform pain self-management, as defined earlier (refer to the Introduction section). The MEDLINE database was searched using a combination of keywords and relevant Medical Subject Headings (MeSH) terms, such as *pain management*, *self-management*, *cancer*, *pain*, *barriers*, *facilitators*, and *patient-related barriers* (refer to Multimedia Appendix 1 for the search strategy). The search was restricted to any type of review article to identify the evidence-based barriers to, and facilitators of, the target behaviors. The identified relevant articles were analyzed to extract all barriers and facilitators relevant to pain self-management. The barriers and facilitators were coded and mapped to the COM-B components. This was represented in a table that served as the foundation for mapping theoretical components to app features.

### Stage 2: Identifying Intervention Options

#### Step 5: Identifying Intervention Functions

There are 9 BCW intervention functions (each function can serve multiple COM-B components, and each component can be served by different functions). They are education, persuasion, incentivization, coercion, training, restriction, environmental restructuring, modeling, and enablement [34,38]. The COM-B components identified in the previous stage were mapped to intervention functions that are likely to serve them and bring about change according to the BCW’s guidelines [38].

#### Step 6: Identifying Policy Categories

The BCW identified 7 policy categories that could effectively support the delivery of the intervention functions and provided a matrix for this process. The possible policy categories are communication and marketing, guidelines, fiscal measures, regulation, legislation, environmental and social planning, and service provision [34,38]. In this study, the intended app was conceptualized as falling in the service provision policy category according to the BCW [34,38]. Therefore, this step was used to refine the candidate intervention functions identified in the previous step to only the functions that could be delivered using this policy category.

Furthermore, the BCW framework emphasizes the importance of considering the context of the intervention as all steps are implemented and selecting what is most appropriate for the intervention to ensure effectiveness [34,38]. In line with this, the affordability, practicability, effectiveness and cost-effectiveness, acceptability, side effects and safety, and equity (APEASE) criteria, suggested by the BCW [38], were applied to the candidate intervention functions. This was to guide the judgment in selecting the most suitable functions that the intervention can serve within its context. The judgment was first made by AAA based on the criteria application; subsequently, it was reviewed by the other authors. The selected functions were then mapped to the original intervention table produced in the first stage.

### Stage 3: Identifying Content and Implementation Options

#### Step 7: Identifying BCTs

The contents of the intervention were identified in this step using the BCTTv1 [25]. This was achieved, in accordance with the BCW guidelines [38], through mapping the selected intervention functions from the previous steps to possible BCTs that are relevant to serve the functions and induce the desired change. The BCW identified a list of candidate BCTs for each intervention function and classified them into most and least frequently used BCTs. Both groups were considered for this intervention. Some BCTs are deemed appropriate for different intervention functions. Web-based training provided by the BCTTv1 developers [47] was taken to help in understanding BCT labels and definitions and in applying the taxonomy accurately and reliably. All candidate BCTs for the intervention were considered with regard to their appropriateness to the context using the APEASE criteria. Next, evidence from the literature on BCTs used in effective interventions was considered to support the final selection of potentially effective and evidence-based BCTs to be incorporated into the app design. The database of BCTTv1-coded interventions [48] was searched for interventions that focused on self-management as the target behavior. There was found to be a lack of interventions and reviews in supporting pain self-management for patients with cancer using the BCTTv1. Therefore, BCTs serving effective interventions supporting self-management in any health condition were included. The selected BCTs were then mapped to the original intervention table with examples given on how these BCTs could be applied in the intervention context. Translating BCTs into app features was not guided by the BCW framework. Digital behavior change interventions [41,43,44,46,49] that used some of the BCTs identified for the app were reviewed to learn how the BCTs could be represented.

#### Step 8: Identifying the Mode of Delivery

The selected mode of delivery, as discussed earlier, is a mobile phone app. Therefore, this step of the framework was not considered.

## Results

### Stage 1: Understanding the Behavior and What Needs to Change

#### Steps 1, 2, and 3: Defining, Selecting, and Specifying the Target Behaviors

##### Step 1

From a behavioral perspective, it was clear from the literature that the problem of unsatisfactory pain management is related to a combination of behaviors on the HCP, health care system, and patient levels [50-52].

##### Step 2

Pain self-management was selected as the target behavior for the app because it shows strong supporting evidence for better pain management [53-58].

##### Step 3

Patients need to self-manage their pain at home and during the period of experiencing pain by incorporating pain control strategies into daily life and communicating with HCPs.

##### Step 4: Identifying What Needs to Change

Five relevant review articles [51,55-58] were generated from the search and used for the behavioral diagnosis. The behavioral diagnosis shown in Multimedia Appendix 2 indicated that physical and psychological capability, physical and social opportunity, and automatic and reflective motivation needed to change for the pain self-management to be performed. Multimedia Appendix 2 serves as the intervention mapping table for the rest of the analysis results.

### Stage 2: Identifying Intervention Options (Steps 5 and 6: Identifying and Refining Intervention Functions)

Mapping the intervention functions to the corresponding COM-B components indicated that all 9 intervention functions were appropriate for addressing the identified determinants for pain self-management behavior (Table 1). However, refining the functions to be delivered through the app (ie, service provision policy category) resulted in the following 7 (78%) of 9 functions being considered for inclusion: education, persuasion, incentivization, coercion, training, modeling, and enablement (Table 1). Moreover, after considering each candidate of the intervention functions using the APEASE criteria, 4 (57%) of the 7 functions were selected: education, persuasion, training, and enablement; the reasons for selecting these are detailed in Table 2. Multimedia Appendix 2 illustrates mapping the selected functions to the previous results.

**Table 1:** Mapping intervention functions to capability, opportunity, motivation, and behavior (COM-B) components with consideration to the selected policy category.

COM-B components	Candidate intervention functions								
	Education	Persuasion	Incentivization	Coercion	Training	Restriction	Environmental restructuring	Modeling	Enablement
Physical capability					✓				✓
Psychological capability	✓				✓				✓
Physical opportunity					✓	✓^a^	✓^a^		✓
Social opportunity						✓^a^	✓^a^	✓	✓
Automatic motivation		✓	✓	✓	✓		✓^a^	✓	✓
Reflective motivation	✓	✓	✓	✓					

^a^Inappropriate intervention function to deliver through service provision policy category.

**Table 2:** Applying the affordability, practicability, effectiveness and cost-effectiveness, acceptability, side effects and safety, and equity (APEASE) criteria to guide the selection of intervention functions.

Candidate intervention functions	Definition [34,38]	Does the intervention function meet the APEASE criteria in the context of using an app to support pain self-management?
Education	“Increasing knowledge or understanding”	Yes
Persuasion	“Using communication to induce positive or negative feelings or stimulate action”	Yes
Incentivization	“Creating an expectation of reward”	Not practicable and unlikely to be effective in this context
Coercion	“Creating an expectation of punishment or cost”	Not acceptable to patients and not practicable to deliver in this context
Training	“Imparting skills”	Yes
Modeling	“Providing an example for people to aspire to or imitate”	Not practicable or relevant to deliver in this context
Enablement	“Increasing means or reducing barriers to increase capability or opportunity”	Yes

### Stage 3: Identifying Content and Implementation Options (Step 7: Identifying BCTs)

A total of 65 candidate BCTs were derived from linking the selected intervention functions to BCTs. This set was refined based on considering the context and applying the APEASE criteria to the 18 BCTs listed in Table 3 (refer to Multimedia Appendix 3 [25] for the full analysis), 15 (83%) of which were found to have been used in effective self-management interventions, as specified in Table 3. Multimedia Appendix 2 outlines how these 18 BCTs were mapped to the previous analysis, along with examples of how they could be represented to bring about change and encourage patients to perform pain self-management.

**Table 3:** Mapping intervention functions to behavior change techniques (BCTs).

Intervention function and BCT label^a^			BCT definition [25]		Evidence for effectiveness
**Education**					
	2.2. Feedback on behavior	“Monitor and provide informative or evaluative feedback on performance of the behavior (eg, form, frequency, duration, and intensity)”		Yes [29,59]	
	2.3. Self-monitoring of behavior	“Establish a method for the person to monitor and record their behavior of behaviors as part of a behavior change strategy”		Yes [29,60]	
	2.7. Feedback on outcome of behavior	“Monitor and provide feedback on the outcome of performance of the behavior”		Yes [60]	
	5.1. Information about health consequences	“Provide information (eg, written, verbal, and visual) about health consequences of performing the behavior”		Yes [29,59,60]	
	5.3. Information about social and environmental consequences	“Provide information (eg, written, verbal, and visual) about social and environmental consequences of performing the behavior”		No	
	7.1. Prompts or cues	“Introduce or define environmental or social stimulus with the purpose of prompting or cueing the behavior”		Yes [60]	
	2.4. Self-monitoring of outcome(s) of behavior^b^	“Establish a method for the person to monitor and record the outcome(s) of their behavior as part of a behavior change strategy”		Yes [29]	
	5.6. Information about emotional consequences^b^	“Provide information (eg, written, verbal, and visual) about emotional consequences of performing the behavior”		No	
	6.3. Information about others’ approval^b^	“Provide information about what other people think about the behavior. The information clarifies whether others will like, approve or disapprove of what the person is doing or will do”		No	
**Persuasion**					
	2.2. Feedback on behavior	“Monitor and provide informative or evaluative feedback on performance of the behavior (eg, form, frequency, duration, and intensity)”		Yes [29,59]	
	2.7. Feedback on outcome(s) of behavior	“Monitor and provide feedback on the outcome of performance of the behavior”		Yes [60]	
	5.1. Information about health consequences	“Provide information (eg, written, verbal, and visual) about health consequences of performing the behavior”		Yes [29,59,60]	
	5.3. Information about social and environmental consequences	“Provide information (eg, written, verbal, and visual) about social and environmental consequences of performing the behavior”		No	
	5.6. Information about emotional consequences^b^	“Provide information (eg, written, verbal, and visual) about emotional consequences of performing the behavior”		No	
	6.3. Information about others’ approval^b^	“Provide information about what other people think about the behavior. The information clarifies whether others will like, approve or disapprove of what the person is doing or will do”		No	
**Training**					
	2.2. Feedback on behavior	“Monitor and provide informative or evaluative feedback on performance of the behavior (eg, form, frequency, duration, and intensity)”		Yes [29,59]	
	2.3. Self-monitoring of behavior	“Establish a method for the person to monitor and record their behavior(s) as part of a behavior change strategy”		Yes [29,60]	
	2.7. Feedback on outcome(s) of behavior	“Monitor and provide feedback on the outcome of performance of the behavior”		Yes [60]	
	4.1. Instruction on how to perform a behavior	“Advise or agree on how to perform the behavior”		Yes [60,61]	
	2.4. Self-monitoring of outcome(s) of behavior^b^	“Establish a method for the person to monitor and record the outcome(s) of their behavior as part of a behavior change strategy”		Yes [29]	
**Enablement**					
	1.2. Problem-solving	“Analyze, or prompt the person to analyze, factors influencing the behavior and generate or select strategies that include overcoming barriers and/or increasing facilitators”		Yes [29,59,61,62]	
	2.3. Self-monitoring of behavior	“Establish a method for the person to monitor and record their behavior(s) as part of a behavior change strategy”		Yes [29,60]	
	3.1. Social support (unspecified)	“Advise on, arrange or provide social support (eg, from friends, relatives, colleagues, ‘buddies,’ or staff) or non-contingent praise or reward for performance of the behavior”		Yes [29]	
	3.2. Social support (practical)	“Advise on, arrange, or provide practical help (eg, from friends, relatives, colleagues, ‘buddies’ or staff) for performance of the behavior”		Yes [60]	
	2.4. Self-monitoring of outcome(s) of behavior^b^	“Establish a method for the person to monitor and record the outcome(s) of their behavior as part of a behavior change strategy”		Yes [29]	
	3.3. Social support (emotional)^b^	“Advise on, arrange, or provide emotional social support (eg, from friends, relatives, colleagues, ‘buddies’ or staff) for performance of the behavior”		Yes [29]	
	11.1. Pharmacological support^b^	“Provide, or encourage the use of or adherence to, drugs to facilitate behavior change”		Yes [29,61,62]	
	11.2. Reduce negative emotions^b^	“Advise on ways of reducing negative emotions to facilitate performance of the behavior”		Yes [29,62]	
	12.2. Restructuring the social environment^b^	“Change, or advise to change, the social environment in order to facilitate performance of the wanted behavior, or create barriers to the unwanted behavior”		Yes [29]	
	12.6. Body changes^b^	“Alter body structure, functioning or support directly to facilitate behavior change”		Yes [61]	

^a^The number beside each BCT label refers to the classification label in the Behavior Change Technique Taxonomy version 1.

^b^Less frequently used BCTs identified for the intervention function.

## Discussion

### Principal Findings

Much of the recent literature on evaluating pain apps has revealed the absence of any theoretical foundation and evidence-based features [16-18]. This study reports the theory- and evidence-driven design of an app, which is intended to support pain self-management, through the application of the BCW framework. This theoretical framework helps explain the mechanisms through which the intervention is likely to influence behavior change [46]. This is in line with the UK MRC recommendation for developing and evaluating complex interventions [12].

In this study, the results of the fundamental phase of the BCW, the behavioral diagnosis based on the COM-B model, revealed that patients may have deficits in their capability, opportunity, and motivation that prevent them from performing pain self-management (Multimedia Appendix 2). Consequently, the determinants derived from the literature in relation to the diagnosis were identified to be targeted by the app. They were in accordance with indicators that have been identified for nurses to assess whether patients with cancer pain can perform pain self-management [30]. These were labeled as physical functions, cognitive abilities, motivation, undergoing treatment for pain, receiving individual education, receiving family and HCPs’ support, and health literacy [30].

The app needs to use education, persuasion, training, and enablement intervention functions because, based on the analysis, they were found the most likely to address the specified factors. Incentivization, coercion, and modeling intervention functions were also suggested by the BCW, but they were excluded (Table 2). This was because the nature and complexity of the disease and the pain do not allow these types of intervention functions to be practicable or acceptable; for example, it would be inappropriate to show any form of reward or punishment simply because pain was controlled or not, respectively. In some cases, a patient’s effort to cope with pain might not be very successful.

Eighteen BCTs were selected to describe specifically how the intervention functions can be presented to induce the desired change regarding the intervention context (Table 3). In other words, they were selected to form the active contents of the app, potentially reducing barriers and serving to support patients in the self-management of pain while using the app, as the context examples illustrate in Multimedia Appendix 2; for example, to increase patients’ motivation, which could be affected by the belief that pain increases as the disease progresses and so cannot be managed, the app can serve to educate patients by explaining the health consequences of cancer, including pain, to correct the misconception. In addition, the app can use a persuasion function, which could be presented through asking patients to monitor and record pain levels. This has the potential to improve self-efficacy through observing positive experiences and trigger problem-solving through noting unsuccessful ones.

The results showed that, of the 18 identified BCTs, 15 (83%) had previously been applied in effective self-management interventions, whereas 3 (17%), namely *information about others’* *approval*, *information about emotional consequences*, and *information about social and environmental consequences*, had no evidence of earlier use. Despite the lack of evidence of the effectiveness to support the exceptions, it was decided to include them in the design of the app to provide evidence about their effectiveness in a future work. Some of the BCTs (38/65, 58%) proposed by the BCW were found inappropriate to the context, such as *biofeedback*, *identification of self as role model*, and *social comparison*. Other promising BCTs (9/65, 14%) cannot be delivered through the app, such as *goal setting*, *review behavior goal*, and *action planning*; these are likely to require nurse coaching to be better implemented, which was beyond the scope of this study (Multimedia Appendix 3); for example, the aforementioned BCTs were successfully implemented by Koller et al [29] in their intervention, which involved coaching nurses to support patients’ pain self-management. In addition, not every face-to-face intervention can be translated to mobile technology, but questioning whether it is possible is important [63].

The BCTs specified for the app need to be carefully translated and implemented as meaningful app features because no guidance is provided by the BCW in relation to this matter. It is crucial that the BCTs are delivered in optimal ways that ensure patients’ engagement; therefore, the user-centered design approach is recommended to be adopted for building the app in line with patients’ preferences. BCTs such as *self-monitoring* and *feedback* will not be effective if patients lose interest in using the app.

It is important to acknowledge that the application of behavior change theory in digital health is still an emerging area of research, with creation of an agenda to guide the development of research only started in recent years [64]. The behavioral intervention technology (BIT) model is another conceptual framework that aims to integrate behavioral science and technology and to support the translation of the behavior change strategies into features of BIT, such as apps [65]. Unlike the BCW framework, the BIT model does not consider understanding the target behavior from the early stages, and it does not provide intervention designers with all possible options for solving the problem; therefore, they can systematically select the most appropriate one for the context. In addition, it does not support the integration of the user-centered design method [43]. Indeed, these aspects were believed to be essential factors for increasing the likelihood of success of mHealth apps [43].

On the basis of the aforementioned particulars, this study provided a step-by-step theory- and evidence-based design for the intended app. Such clarity was considered minimal or even nonexistent in the practice of interventions claiming that they are guided by theory [66]. Characterizing the app by well-defined and evidence-based BCTs might allow replication and easier evidence synthesis regarding the effectiveness of intervention contents, which was difficult to achieve as the evidence suggested [7,8,19-21].

### Limitations

The behavioral analysis was based on literature from only 1 database because the topic of patients’ barriers to cancer pain management seemed well investigated. Nevertheless, searching more databases and additional data from multiple sources, such as focus groups or interviews, could have revealed further insights and strengthened the understanding of cancer pain self-management behaviors. Consequently, this could have resulted in a more precise selection of BCTs and effective interventions. Another limitation was related to implementing the stepwise approach recommended by the BCW team (Figure 1). Although it seems straightforward, it was hard to follow in practice because it involved shifting back and forth among steps as issues were discovered. It required using a great amount of judgment regarding what is most appropriate for the context, which involved consultations with the framework developers as well as with some experts in pain management. This may make it necessary to revisit the earlier stages at times, and this might not be clearly documented.

### Conclusions

There has been increasing emphasis on the need for underpinning theory and evidence for mHealth interventions to ensure their success and facilitate their appraisal. The work in this study demonstrated the application of the BCW framework in designing and developing an app for supporting pain self-management for patients with cancer. The app design will be based on education, persuasion, training, and enablement intervention functions that will be presented by 18 BCTs. To the best of our knowledge, this is the first systematic theory- and evidence-driven design for a pain app for patients with cancer. This systematic approach can support clarity in the evaluation of the underlying mechanisms of the intervention and support future replication.

## Appendix

Multimedia Appendix 1 Search strategy for the behavioral diagnosis.

Multimedia Appendix 2 Behavioral diagnosis for pain self-management using the capability, opportunity, motivation, and behavior (COM-B) model.

Multimedia Appendix 3 Mapping intervention functions to behavior change techniques and applying affordability, practicability, effectiveness and cost-effectiveness, acceptability, side effects and safety, and equity (APEASE) criteria.

## Abbreviations

APEASE: affordability, practicability, effectiveness and cost-effectiveness, acceptability, side effects and safety, and equity
BCT: behavior change technique
BCTTv1: Behavior Change Technique Taxonomy version 1
BCW: Behavior Change Wheel
BIT: behavioral intervention technology
COM-B: capability, opportunity, motivation, and behavior
HCP: health care professional
MeSH: Medical Subject Headings
mHealth: mobile health
MRC: Medical Research Council

## Data Availability

Data collected in this work are presented within the manuscript and the supplementary documents.
