# Supplementary material for: Supporting Pain Self-Management in Patients With Cancer: App Development Based on a Theoretical and Evidence-Driven Approach
Source: JMIR Cancer. 2023 Oct 9;9:e49471. doi: 10.2196/49471 (PMC10594136; doi:10.2196/49471)
Supplement: Multimedia Appendix 1 [file cancer_v9i1e49471_app1.docx]

Multimedia Appendix 1: Search strategy for the behavioral diagnosis

| **Search #** | **Search terms (number of records)** |
| --- | --- |
| 1 | cancer.mp. or exp Neoplasms/ (2093540) |
| 2 | 'patient-related barriers '.mp. (123) |
| 3 | patient.mp. [mp=title, abstract, original title, name of substance word, subject heading word, floating sub-heading word, keyword heading word, protocol supplementary concept word, rare disease supplementary concept word, unique identifier, synonyms] (1680366) |
| 4 | barrier.mp. [mp=title, abstract, original title, name of substance word, subject heading word, floating sub-heading word, keyword heading word, protocol supplementary concept word, rare disease supplementary concept word, unique identifier, synonyms] (103661) |
| 5 | facilitators.mp. [mp=title, abstract, original title, name of substance word, subject heading word, floating sub-heading word, keyword heading word, protocol supplementary concept word, rare disease supplementary concept word, unique identifier, synonyms] (8335) |
| 6 | 4 or 5 (111463) |
| 7 | 3 and 6 (11068) |
| 8 | 2 or 7 (11159) |
| 9 | self management.mp. or exp Self Care/ or exp Self-Management/ (44869) |
| 10 | pain.mp. or exp PAIN/ (468849) |
| 11 | 9 and 10 (3145) |
| 12 | pain management.mp. or exp Pain Management/ (30638) |
| 13 | 11 or 12 (33134) |
| 14 | 1 and 8 and 13 (81) |
| 15 | limit 14 to (english language and "review articles") (15) |
