# Supplementary material for: Supporting Pain Self-Management in Patients With Cancer: App Development Based on a Theoretical and Evidence-Driven Approach
Source: JMIR Cancer. 2023 Oct 9;9:e49471. doi: 10.2196/49471 (PMC10594136; doi:10.2196/49471)
Supplement: Multimedia Appendix 2 [file cancer_v9i1e49471_app2.docx]

Multimedia Appendix 2. Behavioral diagnosis for pain self-management using the capability, opportunity, motivation, and behavior (COM-B) model.

| COM-B components | | Barriers and facilitators of pain self-management | Intervention functions | BCT^a^ labels^b^ | | Examples of application (BCT) |
| --- | --- | --- | --- | --- | --- | --- |
| **Capability** | | | | | | |
|  | Physical | - Barrier: sensory barriers such as side effects of pain medication [55] - Barrier: lack of skills regarding how to manage different patterns and types of pain [56] - Facilitator: being able to use nonpharmacological strategies [56] | - Training - Enablement | - 4.1. Instruction on how to perform a behavior - 1.2. Problem-solving - 12.6. Body changes | | - Provide patients with information and instructions about pain and pain control strategies (4.1) - Using the app itself to record pain details over time has the potential to trigger problem-solving (1.2) - Provide patients with information about how to physically cope with cancer and medication side effects, such as the benefit of routine physical exercise (4.1 and 12.6) |
|  | Psychological | - Barrier: poor knowledge about pain medication and their side effects [55,57] - Barrier: lack of awareness of which services and management options are available and how to manage pain at home [56] - Barrier: lack of skills regarding how to manage different patterns and types of pain [56] - Facilitator: being educated about the disease, pain and pain medication will facilitate changing knowledge, attitudes, and beliefs [51,56,58] - Facilitator: being aware of nonpharmacological strategies [56] | - Education - Training - Enablement | - 2.2. Feedback on behavior - 2.3. Self-monitoring of behavior - 2.7. Feedback on outcome of behavior - 5.1. Information about health consequences - 7.1. Prompts and cues - 2.4. Self-monitoring of outcome(s) of behavior - 6.3. Information about others’ approval - 5.3. Information about social and environmental consequences - 5.6. Information about emotional consequences - 4.1. Instruction on how to perform a behavior - 11.1. Pharmacological support | | - Inform patients about support services available for them and about nonpharmacological pain control strategies, and remind them to consider using them (4.1 and 7.1) - Inform patients about pain and pain medications and their effects (5.1 and 11.1) - Ask patients to record daily their adherence to medication as prescribed and whether they have used any other pain control strategy; provide them with a form for recording this and a method for reviewing it (2.3 and 2.4) - Inform patients that HCPs^c^ approve conscious patients who self-manage their pain and keep partnership with them (6.3) |
| **Opportunity** | | | | | | |
|  | Physical | - Barrier: no access to nearby specialist services and the need to travel with pain and being away from family and work [56] - Facilitator: being able to record pain and medication in diaries [51] | - Training - Enablement | | - 2.3. Self-monitoring of behavior - 2.4. Self-monitoring of outcome(s) of behavior - 1.2. Problem-solving - 3.1. Social support (unspecified) - 3.2. Social support (practical) | - The app functions as an electronic diary and facilitates reporting pain to HCPs and identifying nearby support services (2.3, 2.4, 1.2, 3.1, and 3.2) |
|  | Social | - Barrier: cultural norms regarding experience of cancer pain and attitudes toward its management [56] - Facilitator: developing trust in HCPs makes patients more likely to report pain [56] | - Enablement | | - 2.3. Self-monitoring of behavior - 2.4. Self-monitoring of outcome(s) of behavior - 3.1. Social support (unspecified) - 3.2. Social support (practical) - 3.3. Social support (emotional) - 12.2. Restructuring the social environment | - The app provides patients with ways of getting social support, such as chatting with other patients and identifying nearby social support services (3.1, 3.2, 3.3, and 12.2) - The app facilitates reporting pain to HCPs, thus improving the partnership between the patient and the HCP (2.3 and 2.4) |
| **Motivation** | | | | | | |
|  | Automatic | - Barrier: cognitive barriers to pain medication, including fear of addiction, side effects, tolerance, and being harmful for immune system [51,55-58] - Barrier: mood disorders and emotional barriers, such as depression and mood fluctuations [51,55] - Barrier: anxiety from the uncertainty and fear that the pain will get worse [56] | - Persuasion - Training - Enablement | - 5.1. Information about health consequences - 5.3. Information about social and environmental consequences - 5.6. Information about emotional consequences - 11.1. Pharmacological support - 11.2. Reduce negative emotions | | - Explain to patients that self-management of pain through adherence to prescribed medication and adopting a pain control strategy can help in preventing pain deterioration and unscheduled visits to hospitals and lower the financial burden on the health care system, increasing the feeling of being in control of their condition and well-being (5.1, 5.3, 5.6, and 11.1) - Advise patients on ways of reducing negative emotions (11.2) |
|  | Reflective | - Barrier: patients are reluctant to adhere to treatment recommendations [55,57,58] - Barrier: belief that “good patients” do not complain about pain and should be stoic and endure pain [51,55,56,58] - Barrier: belief that reporting pain distracts HCP from treating cancer [51,55,56,58] - Barrier: perceived lack of trust in health care system and HCPs [55,56] - Barrier: belief that increased pain indicates a progression of the cancer and that cancer pain is inevitable and cannot be managed [51,55,56,58] | - Education - Persuasion | - 2.2. Feedback on behavior - 2.3. Self-monitoring of behavior - 2.7. Feedback on outcome(s) of behavior - 5.1. Information about health consequences - 7.1. Prompts/cues - 2.4. Self-monitoring of outcome(s) of behavior - 6.3. Information about others’ approval - 5.3. Information about social and environmental consequences - 5.6. Information about emotional consequences | | - Inform patients that HCPs approve of keeping a good partnership with them; this helps HCPs to build a full pain record for better pain assessment and intervene more effectively at the right time, preventing pain deterioration, unscheduled visits to hospitals, and burden on health care system (6.3, 5.1, and 5.3) - Ask patients to report daily and record their pain level and provide them with some feedback about the pain pattern; in addition, provide them with a method for measuring pain and recording its scores as well as a method for reviewing it (2.2, 2.3, 2.4, and 2.7) - Remind patients to record pain consistently and maintain the partnership with their HCPs (7.1) |

^a^BCT: behavior change technique.

^b^The number beside each BCT label refers to the classification label in the Behavior Change Technique Taxonomy version 1.

^c^HCP: health care professional.
