# Supplementary material for: Supporting Pain Self-Management in Patients With Cancer: App Development Based on a Theoretical and Evidence-Driven Approach
Source: JMIR Cancer. 2023 Oct 9;9:e49471. doi: 10.2196/49471 (PMC10594136; doi:10.2196/49471)
Supplement: Multimedia Appendix 3 [file cancer_v9i1e49471_app3.docx]

Multimedia Appendix 3: Mapping intervention functions to BCTs and applying APEASE criteria*

| **Intervention function** | **BCT label*** | **BCT Definition [25]** | **Does the BCT meet the APEASE criteria in the context of using an app to support pain self-management?** |
| --- | --- | --- | --- |
| Education | 2.2. Feedback on behaviour | “Monitor and provide informative or evaluative feedback on performance of the behaviour (e.g. form, frequency, duration, intensity)” | Yes |
|  | 2.3. Self-monitoring of behaviour | “Establish a method for the person to monitor and record their behaviour(s) as part of a behaviour change strategy” | Yes |
|  | 2.7. Feedback on outcome(s) of behaviour | “Monitor and provide feedback on the outcome of performance of the behaviour” | Yes |
|  | 5.1. Information about health consequences | “Provide information (e.g. written, verbal, visual) about health consequences of performing the behaviour” | Yes |
|  | 5.3. Information about social and environmental consequences | “Provide information (e.g. written, verbal, visual) about social and environmental consequences of performing the behaviour” | Yes |
|  | 7.1. Prompts/cues | “Introduce or define environmental or social stimulus with the purpose of prompting or cueing the behaviour” | Yes |
|  | 2.6. Biofeedback | “Provide feedback about the body (e.g. physiological or biochemical state) using an external monitoring device as part of a behaviour change strategy” | Not practicable or relevant in this context |
|  | 2.4. Self-monitoring of outcome(s) of behaviour | “Establish a method for the person to monitor and record the outcome(s) of their behaviour as part of a behaviour change strategy” | Yes |
|  | 7.2. Cue signalling reward | “Identify an environmental stimulus that reliably predicts that reward will follow the behaviour (includes ‘Discriminative cue’)” | Not practicable or relevant in this context |
|  | 7.6. Satiation | “Advise or arrange repeated exposure to a stimulus that reduces or extinguishes a drive for the unwanted behaviour” | Not relevant in this context |
|  | 4.2. Information about antecedents | “Provide information about antecedents (e.g. social and environmental situations and events, emotions, cognitions) that reliably predict performance of the behaviour” | Not relevant in this context |
|  | 4.3. Re-attribution | “Elicit perceived causes of behaviour and suggest alternative explanations (e.g. external or internal and stable or unstable)” | Not practicable or relevant in this context |
|  | 4.4. Behavioural experiments | “Advise on how to identify and test hypotheses about the behaviour, its causes and consequences, by collecting and interpreting data” | Not practicable or relevant in this context |
|  | 5.6. Information about emotional consequences | “Provide information (e.g. written, verbal, visual) about emotional consequences of performing the behaviour” | Yes |
|  | 6.3. Information about others’ approval | “Provide information about what other people think about the behaviour. The information clarifies whether others will like, approve or disapprove of what the person is doing or will do” | Yes |
| Persuasion | 2.2. Feedback on behaviour | Previously defined | See above |
|  | 2.7. Feedback on outcome(s) of behaviour | Previously defined | See above |
|  | 5.1. Information about health consequences | Previously defined | See above |
|  | 5.3. Information about social and environmental consequences | Previously defined | See above |
|  | 9.1. Credible source | “Present verbal or visual communication from a credible source in favour of or against the behaviour” | Not practicable to deliver in this context |
|  | 2.6. Biofeedback | Previously defined | See above |
|  | 4.3. Re-attribution | Previously defined | See above |
|  | 5.2. Salience of consequences | “Use methods specifically designed to emphasise the consequences of performing the behaviour with the aim of making them more memorable” | Not practicable or relevant in this context |
|  | 5.6. Information about emotional consequences | Previously defined | See above |
|  | 6.2. Social comparison | “Draw attention to others’ performance to allow comparison with the person’s own performance” | Not practicable and acceptable to deliver in this context |
|  | 6.3. Information about others’ approval | Previously defined | See above |
|  | 13.1. Identification of self as role model | “Inform that one's own behaviour may be an example to others” | Not relevant in this context |
|  | 13.2. Framing/reframing | “Suggest the deliberate adoption of a perspective or new perspective on behaviour (e.g. its purpose) in order to change cognitions or emotions about performing the behaviour” | Not practicable or relevant in this context |
|  | 13.5. Identity associated with changed behaviour | “Advise the person to construct a new self-identity as someone who ‘used to engage with the unwanted behaviour’” | Unlikely to be acceptable and effective in this context |
|  | 15.1. Verbal persuasion about capability | “Tell the person that they can successfully perform the wanted behaviour, arguing against self-doubts and asserting that they can and will succeed” | Not practicable or relevant in this context |
|  | 15.3. Focus on past success | “Advise to think about or list previous successes in performing the behaviour (or parts of it)” | Not practicable or relevant in this context |
| Training | 2.2. Feedback on behaviour | Previously defined | See above |
|  | 2.3. Self-monitoring of behaviour | Previously defined | See above |
|  | 2.7. Feedback on outcome(s) of behaviour | Previously defined | See above |
|  | 4.1. Instruction on how to perform a behaviour | “Advise or agree on how to perform the behaviour” | Yes |
|  | 6.1. Demonstration of the behaviour | “Provide an observable sample of the performance of the behaviour, directly in person or indirectly e.g. via film, pictures, for the person to aspire to or imitate” | Not practicable or relevant in this context |
|  | 8.1. Behavioural practice/rehearsal | “Prompt practice or rehearsal of the performance of the behaviour one or more times in a context or at a time when the performance may not be necessary, in order to increase habit and skill” | Not practicable or relevant in this context |
|  | 2.4. Self-monitoring of outcome(s) of behaviour | Previously defined | See above |
|  | 2.6. Biofeedback | Previously defined | See above |
|  | 4.4. Behavioural experiments | Previously defined | See above |
|  | 8.3. Habit formation | “Prompt rehearsal and repetition of the behaviour in the same context repeatedly so that the context elicits the behaviour” | Unlikely to be acceptable and effective in this context |
|  | 8.4. Habit reversal | “Prompt rehearsal and repetition of an alternative behaviour to replace an unwanted habitual behaviour” | Not relevant in this context |
|  | 8.7. Graded tasks | “Set easy-to-perform tasks, making them increasingly difficult, but achievable, until behaviour is performed” | Not relevant in this context |
|  | 10.9. Self-reward | “Prompt self-praise or self-reward if and only if there has been effort and/or progress in performing the behaviour” | Unlikely to be acceptable and effective in this context |
|  | 15.4. Self-talk | “Prompt positive self-talk (aloud or silently) before and during the behaviour” | Not practicable or relevant in this context |
|  | 15.2. Mental rehearsal of successful performance | “Advise to practise imagining performing the behaviour successfully in relevant contexts” | Unlikely to be acceptable and effective in this context |
| Enablement | 1.1. Goal setting (behaviour) | “Set or agree on a goal defined in terms of the behaviour to be achieved” | Not practicable or relevant in this context |
|  | 1.2. Problem solving | “Analyse, or prompt the person to analyse, factors influencing the behaviour and generate or select strategies that include overcoming barriers and/or increasing facilitators” | Yes |
|  | 1.3. Goal setting (outcome) | “Set or agree on a goal defined in terms of a positive outcome of wanted behaviour” | Not practicable or relevant in this context |
|  | 1.4. Action planning | “Prompt detailed planning of performance of the behaviour (must include at least one of context, frequency, duration and intensity)” | Not practicable or relevant in this context |
|  | 1.5. Review behaviour goal(s) | “Review behaviour goal(s) jointly with the person and consider modifying goal(s) or behaviour change strategy in light of achievement” | Not practicable or relevant in this context |
|  | 1.7. Review outcome goal(s) | “Review outcome goal(s) jointly with the person and consider modifying goal(s) in light of achievement” | Not practicable or relevant in this context |
|  | 2.3. Self-monitoring of behaviour | Previously defined | See above |
|  | 3.1. Social support (unspecified) | “Advise on, arrange or provide social support (e.g. from friends, relatives, colleagues,’ buddies’ or staff) or non-contingent praise or reward for performance of the behaviour” | Yes |
|  | 3.2. Social support (practical) | “Advise on, arrange, or provide practical help (e.g. from friends, relatives, colleagues, ‘buddies’ or staff) for performance of the behaviour” | Yes |
|  | 12.1. Restructuring the physical environment | “Change, or advise to change the physical environment in order to facilitate performance of the wanted behaviour or create barriers to the unwanted behaviour” | Not relevant in this context |
|  | 12.5. Adding objects to the environment | “Add objects to the environment in order to facilitate performance of the behaviour” | Not relevant in this context |
|  | 1.6. Discrepancy between current behaviour and goal | “Draw attention to discrepancies between a person’s current behaviour (in terms of the form, frequency, duration, or intensity of that behaviour) and the person’s previously set outcome goals, behavioural goals or action plans (goes beyond self-monitoring of behaviour)” | Not practicable or relevant in this context |
|  | 1.8. Behavioural contract | “Create a written specification of the behaviour to be performed, agreed on by the person, and witnessed by another” | Not practicable and unlikely to be acceptable in this context |
|  | 1.9. Commitment | “Ask the person to affirm or reaffirm statements indicating commitment to change the behaviour” | Not practicable and unlikely to be effective in this context |
|  | 2.4. Self-monitoring of outcome(s) of behaviour | Previously defined | See above |
|  | 3.3. Social support (emotional) | “Advise on, arrange, or provide emotional social support (e.g. from friends, relatives, colleagues, ‘buddies’ or staff) for performance of the behaviour” | Yes |
|  | 4.4. Behavioural experiments | Previously defined | See above |
|  | 5.2. Salience of consequences | Previously defined | See above |
|  | 5.4. Monitoring of emotional consequences | “Prompt assessment of feelings after attempts at performing the behaviour” | Not practicable or relevant in this context |
|  | 5.5. Anticipated regret | “Induce or raise awareness of expectations of future regret about performance of the unwanted behaviour” | Not practicable and unlikely to be effective in this context |
|  | 8.2. Behaviour substitution | “Prompt substitution of the unwanted behaviour with a wanted or neutral behaviour” | Not relevant in this context |
|  | 8.5. Overcorrection | “Ask to repeat the wanted behaviour in an exaggerated way following an unwanted behaviour” | Not relevant in this context |
|  | 8.6. Generalisation of a target behaviour | “Advise to perform the wanted behaviour, which is already performed in a particular situation, in another situation” | Not relevant in this context |
|  | 8.7. Graded tasks | Previously defined | See above |
|  | 9.2. Pros and cons | “Advise the person to identify and compare reasons for wanting (pros) and not wanting to (cons) change the behaviour” | Not practicable and unlikely to be effective in this context |
|  | 9.3. Comparative imagining of future outcomes | “Prompt or advise the imagining and comparing of future outcomes of changed versus unchanged behaviour” | Not practicable or relevant in this context |
|  | 10.9. Self-reward | Previously defined | See above |
|  | 11.1. Pharmacological support | “Provide, or encourage the use of or adherence to, drugs to facilitate behaviour change” | Yes |
|  | 11.2. Reduce negative emotions | “Advise on ways of reducing negative emotions to facilitate performance of the behaviour” | Yes |
|  | 11.3. Conserve mental resources | “Advise on ways of minimising demands on mental resources to facilitate behaviour change” | Not practicable or relevant in this context |
|  | 12.2. Restructuring the social environment | “Change, or advise to change the social environment in order to facilitate performance of the wanted behaviour or create barriers to the unwanted behaviour” | Yes |
|  | 12.3. Avoidance/reducing exposure to cues for the behaviour | “Advise on how to avoid exposure to specific social and contextual/physical cues for the behaviour, including changing daily or weekly routines” | Not relevant in this context |
|  | 12.4. Distraction | “Advise or arrange to use an alternative focus for attention to avoid triggers for unwanted behaviour” | Not relevant in this context |
|  | 12.6. Body changes | “Alter body structure, functioning or support directly to facilitate behaviour change” | Yes |
|  | 13.1. Identification of self as role model | Previously defined | See above |
|  | 13.2. Framing/reframing | Previously defined | See above |
|  | 13.3. Incompatible beliefs | “Draw attention to discrepancies between current or past behaviour and self-image, in order to create discomfort” | Not practicable or relevant in this context |
|  | 13.4. Valued self-identity | “Advise the person to write or complete rating scales about a cherished value or personal strength as a means of affirming the person’s identity as part of a behaviour change strategy” | Not practicable or relevant in this context |
|  | 13.5. Identity associated with changed behaviour | Previously defined | See above |
|  | 15.1. Verbal persuasion about capability | Previously defined | See above |
|  | 15.2. Mental rehearsal of successful performance | Previously defined | See above |
|  | 15.3. Focus on past success | Previously defined | See above |
|  | 15.4. Self-talk | Previously defined | See above |
|  | 16.1. Imaginary punishment | “Advise to imagine performing the unwanted behaviour in a real-life situation followed by imagining an unpleasant consequence” | Unlikely to be acceptable and effective in this context |
|  | 16.2. Imaginary reward | “Advise to imagine performing the wanted behaviour in a real-life situation followed by imagining a pleasant consequence” | Not practicable and unlikely to be acceptable in this context |
|  | 16.3. Vicarious consequences | “Prompt observation of the consequences (including rewards and punishments) for others when they perform the behaviour” | Not practicable in this context |

* Numbering mentioned beside the BCT’s labels refers to the classification labels in the BCTTv1

* Shaded box = less frequently used BCTs identified for the intervention functions
